# Community-Engaged Research for the Promotion of Healthy Urban Environments: A Case Study of Community Garden Initiative in Shanghai, China

**DOI:** 10.3390/ijerph16214145

**Published:** 2019-10-28

**Authors:** Huaiyun Kou, Sichu Zhang, Yuelai Liu

**Affiliations:** 1 College of Architecture and Urban Planning, Institute for Advanced Study in Architecture and Urban-Rural Planning, Key Laboratory of Ecology and Energy-Saving Study of Dense Habitat of Ministry of Education, Tongji University, Shanghai 200092, China; khy@tongji.edu.cn; 2 Shanghai Urban Construction Vocational College, Shanghai 200433, China; zhangsichu@succ.edu.cn; 3 College of Architecture and Urban Planning, Key Laboratory of Ecology and Energy-Saving Study of Dense Habitat of Ministry of Education, Tongji University, Shanghai 200092, China

**Keywords:** community garden, healthy environment, community-university partnerships, community-engaged research, community building

## Abstract

The importance of community gardens in a healthy urban environment has been extensively documented, while the garden building involving communities has not been much explored in fast-developing cities. This study examines community engagement in garden building activities in a rapid urbanization context, aiming to explore the application of community-engaged research methods for the promotion of neighbourhood environments. The Community Garden Initiative consisting of an array of progressive actions is formulated by the research team, featuring a process of increasing involvement of community members and decreasing intensity of external interventions. These activities have been launched based on community-university partnerships in Shanghai since 2014, synchronising with a transformation of urban regeneration paradigm in China where people-oriented approaches are more emphasized. Five actions covering 70 community gardens are analysed through surveys on participants’ attitudes and perceptions towards the activities. The results of the study presented people’s rapid acceptance of participation in public affairs, reflected possible measures to promote public participation, and confirmed the positive impacts of the garden building on the neighbourhood environmental health as well as on the community-building. Taking into account that residents generally lack the consciousness and capacities required to implement actions at the initial stage of community engagement, we proposed in the conclusion to start with external interventions and capacity buildings carried out by professionals as a supplement to the ‘community-driven’ principle of CBPR methods.

## 1. Introduction

Community gardens, a type of urban green space managed by residents of a neighbourhood, have gained recognition for their positive impacts on public health and neighbourhood cohesion [1,2,3,4]. Previous research has examined the influence of community gardens on local ecology and sustainability [5,6,7,8], residents’ physical and mental health [9,10,11], as well as community empowerment and environmental justice [12,13,14,15,16]. Organisations as diverse as government departments, universities, and NGOs have promoted, though often from different perspectives, the development and construction of community gardens [17,18]. However, current research on community gardens, and their impacts on psychological health and community building in particular, is mostly situated in developed countries [19]. The coverage of worldwide samples is therefore limited, considering spatial and cultural differences between societies [20].

In China, community gardens that involve citizens in their construction and management started to appear only this decade [21]. Public green space was mainly provided by either the city governments or developers. Meanwhile, large-scale demolition and reconstruction as conventional urban renewal models have led to the severe shrink of public space and reduce in neighbourhood communications [22]. Shanghai, which is a representative of high-density and rapid-developing cities, has taken the lead in reforms to seek a way to achieve sustainable urban growth since 2010 [23]. A serious of documents have been released on mobilising citizens’ initiatives to participate in creating a healthy environment. The policy of *Implementation Measures of Shanghai Urban Regeneration,* proposing people-oriented approaches to improve public space and neighbourhood revitalization, marked a transformation of urban regeneration paradigm from the expansion of urban land to the improvement of the quality and efficiency of land use in old urban areas [24,25]. The sub-district governments and neighbourhood committees have launched various improvements to the physical environment through governance innovation, including residential entrances, neighbourhood plazas, balcony greening, murals, etc. The term ‘micro-regeneration’ was coined to refer to these types of small-scale, community-involved, and step-by-step public space regeneration [26].

We, the research team, have launched the Community Garden Initiative in more than sixty communities in Shanghai through community-university partnerships since 2014. This community-engaged experiment aims to improve neighbourhood environments and residents’ health through collaborative horticultural activities [27]. In the transition of community policy from a government-led pattern to a self-governing one [28], participants soon ignited a fever for taking part in environmental regeneration in old neighbourhoods.

In this study, we focus on exploring the application of community-engaged research methods in the garden building domain. Community involvement has been carried out in practice in fast-urbanising areas, but such areas have received limited coverage in theoretical research. To the best of our knowledge, only a few studies have employed community-engaged research methodologies, and these studies generally focused on medicine and social works [29]. Articles about the community garden in China mainly present cases from the landscape perspectives or merely advocate community-based implementation approaches. The outcomes of community-engaged research worldwide lay a foundation for this study. Sherry Arnstein’s Ladder of Citizen Participation provides a basis for evaluating the extent of the community’s involvement in such cases [30]. The National Institute of Environmental Health Sciences (NIEHS) endorses six principles for effective community-based participatory research (CBPR): (1) promoting active collaboration and participation at every stage; (2) fostering co-learning between researchers and participants; (3) ensuring projects are community-driven; (4) disseminating results in useful terms; (5) ensuring research and intervention strategies are culturally appropriate; and (6) defining community as a unit of identity [31]. The community-university partnerships methods that emphasize the shift from one-way university-to-community service to more interactive one between the two, provide the implementation of this study with references for organisation, communication and cooperation [32,33,34]. Other achievements regarding research design, distinguishing common concerns, key interventions, and research ethics guided the research process [35,36,37,38,39].

This study took place over a period of five years and was synchronised with the transformation of urban regeneration, reflecting the rapid development of people’s acceptance of participation in public affairs in China. This study not only delivers references for the growing field of community-based research and practices related to neighbourhood regeneration, but it also provides international research with rich samples and valuable analyses of community participation in healthy environment-building in cities undergoing rapid urbanisation. Meanwhile, the long-time expansion, multi-case, and progressive design of the research also contribute to the design and application of participatory research methodologies.

In this article, we analysed five actions that represented a progressive process in which external interventions were gradually reduced and powers of self-governance were gradually increased. We conducted questionnaire and interview surveys regarding participants’ attitudes and feelings towards the community garden activities. In conclusion, taking into account that residents generally lack the consciousness and capacities required to implement actions at the initial stage of community engagement in fast-urbanising cities, we proposed to start with external interventions by professionals to carry out capacity buildings as a supplement to the ‘community-driven’ principle of CBPR methods. We also confirmed the positive impacts of the garden building on the neighbourhood environmental health as well as to the community-building. 

## 2. Materials and Methodology 

### 2.1. Community-Engaged Approach Based on Community-University Partnerships

We carried out this research on the basis of community-university partnerships. Researchers from the Landscape Architecture Department at T University have collaborated with neighbourhoods mainly in Shanghai to conduct community garden experiments. Most neighbourhoods have neither the initiative to organise such activities nor the technology for landscape projects; thus, they require professional support to both the organisation and technology assistance of the projects. Meanwhile, universities need locations to instigate practice for the instruction of students and citizens, to conduct research, and to transform research into verifiable outcomes. These complementary motivations facilitate cooperation between communities and universities.

We employed multiple cooperative methods of community-university partnerships in the implementation process of the Community Garden Initiative from June 2014 to June 2019, including service cooperation through instructions and consultations, teaching cooperation through service-learning and community training, and researching cooperation through CBPR methods [40].

### 2.2. A Strategy with Progressive Actions of the Community Garden Initiative

We adopted a strategy which divided the Initiative into progressive actions with increasing extent of communities’ involvement and decreasing intensity of external interventions for each step. We formed a framework of five phases: first, launching an experimental community garden in a neighbourhood with the support from the government; second, introducing more groups of participants such as enterprises and NGOs; third, facilitating to expand neighbourhoods coverage of the Initiative in the city; fourth, encouraging residents to organize garden building by themselves; fifth, promoting and spreading community gardens to the whole country.

We conducted community garden activities mainly in old residential neighbourhoods constructed before 2000, where the relationships between residents are normally closer than that in new ones. Meanwhile, the quality of green space in these neighbourhoods are relatively poor and residents’ demands for improving their living environment are high. However, new neighbourhoods or other types of communities are not excluded. We identified sample neighbourhoods through consultations with Sub-district governments and resident committees.

### 2.3. Five Representative Actions

#### 2.3.1. Researchers’ Initiatives with Community Involvement: Herb Garden

**Figure 1:**
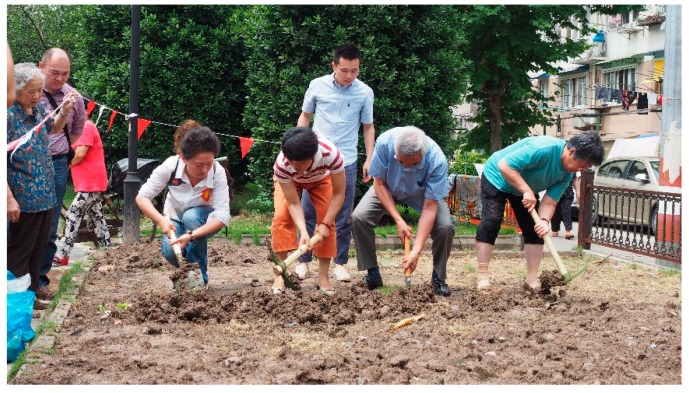
Residents are learning to cultivate soil in the Herb Garden.

The Herb Garden, built in 2014, represents the first case of Community Garden Initiative. The project aimed to transform a monotonic central green space into a shared space where residents could be productive and enjoy nature through participation in landscaping and gardening. The Herb Garden is located in an old neighbourhood next to T University and comprises an area of 210 square metres. This high-density residential neighbourhood was first established in the 1950s with little public space and a high rate of the ageing population at present, but the relationships between residents are harmonious and sound where the general demographics remains stable. The neighbourhood committee also functions well. Many seniors in the neighbourhood who are interested in gardening had already formed a self-governing horticulture group. After holding discussions with the neighbourhood committee, we selected a barren plot that has relatively little impact on residents to implement the garden construction. The design and construction of the garden were mainly conducted by designers in the research team after discussions with residents. The garden contains three areas for leisure activities, parent-child interactions, and nature education. The research group sought residents’ advice after the draft plan was completed. The team also organised ‘Young Landscape Designer’ activities to provide children with opportunities to express their visions and expectations. The construction procedure was separated into several steps, including shaping terrain, cultivating soil, sodding, planting, seeding, paving, and covering the bed. Residents could participate in construction while having access to trainings from the professionals (Figure 1). Residents were also encouraged to bring their own plants to the garden to share with others.

The process of building a community garden enhanced the cohesiveness of the community. By holding various educational activities on nature and other community-building activities related to the Herb Garden, we identified active and capable residents who could act as community leaders to mobilize others’ participation. Two self-governed teams now manage the Herb Garden: the seniors’ horticulture team and the young volunteer team. The horticulture team conduct maintenance through a match of the members’ skills with the demands at different maintenance phases; for example, they have formed groups in charge of different duties such as watering and fertilising, garbage collection, and weeding. These team members also exchange experience in maintenance and management with other residents. The young volunteer team has more than 40 members who conduct simple maintenance tasks in the Herb Garden, such as creating raised beds for vegetables and watering and fertilising plants. They have all played an important role in community-building and garden maintenance. After developing the Herb Garden, we launched community garden building activities by the same approach in another 45 communities in central districts of Shanghai.

#### 2.3.2. Co-construction by Enterprises, Non-Governmental Organisations, and Residents: KIC Garden

**Figure 2:**
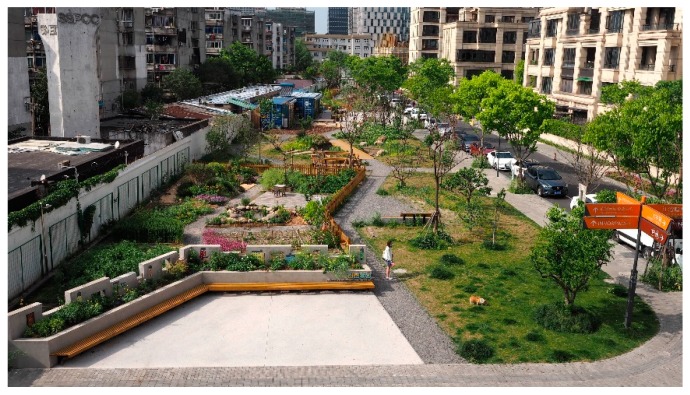
The KIC Garden.

**Figure 3:**
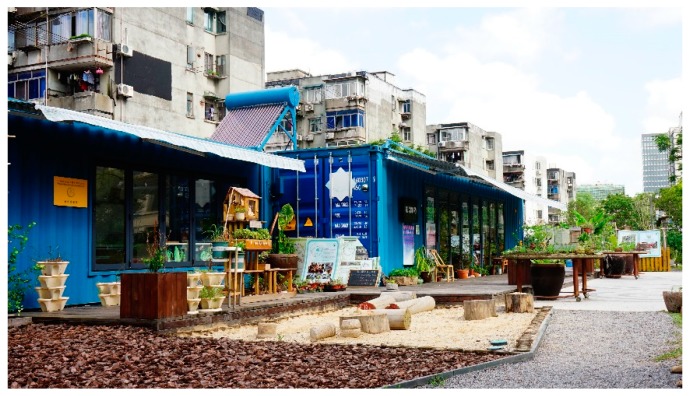
The movable structure in the middle of KIC Garden.

After two years of practice, the Community Garden Initiative formed a certain broader social influence in Shanghai. Companies and non-governmental organisations joined the residents in the building of community gardens. A typical case is the Knowledge and Innovation Community Garden (KIC Garden), which is located between an existing gated neighbourhood (on the left of Figure 2) and a new open neighbourhood (on the right of Figure 2). With an area of 2200 square metres, the narrow lot outside the gated community’s wall was previously vacant because an important municipal tunnel passed underneath. KIC Garden is composed of a public activity area, a permaculture garden, the Square-Metre vegetation garden, and an interaction area. The garden features sustainable energy recycling facilities such as garbage recycling trashcans, an earthworm tower, a composter, and a small greenhouse. A movable structure made from three cargo containers sits in the middle of the garden and is used as an indoor activity space (Figure 3). The new neighbourhood’s property management agency funded the garden, and a non-government organisation has taken part in garden‘s management and operation by engaging residents in its daily maintenance, public science education, and other community empowerment activities. Universities close to KIC Garden provide intellectual resources such as organising academic workshops, nature education, and community concerts. KIC Garden has built a platform encompassing all generations from multiple backgrounds to facilitate exchange and improve environmental health and justice in this culturally diverse city.

**Figure 4:**
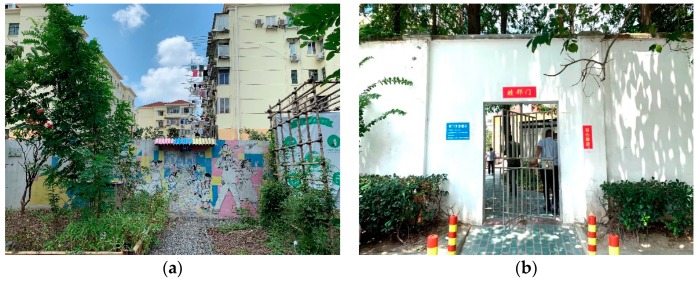
The magic door (**a**) Painting on the wall (**b**) The Door of Harmony.

Previously, due to the separation caused by the wall, residents had to take a detour to attend activities in KIC Garden. During a community art activity initiated by the designers, residents painted a magic door on the wall, with a path leading towards this door in the hopes that future residents would be able to open the door and shorten the distance between KIC Garden and the old neighbourhood (Figure 4a). 

After discussions with the relevant governmental departments, they created a door nearby called the ‘Door of Harmony’ to break the spatial separation between the new and old communities, facilitating interactions between the residents (Figure 4b). With the wish to break down ‘wall in minds’, this community-university cooperative project was selected as one of the ten best social governing innovation practice in China in 2018.

#### 2.3.3. Fostering Community Leaders to Drive Community Participation: Puxing Sub-District

**Figure 5:**
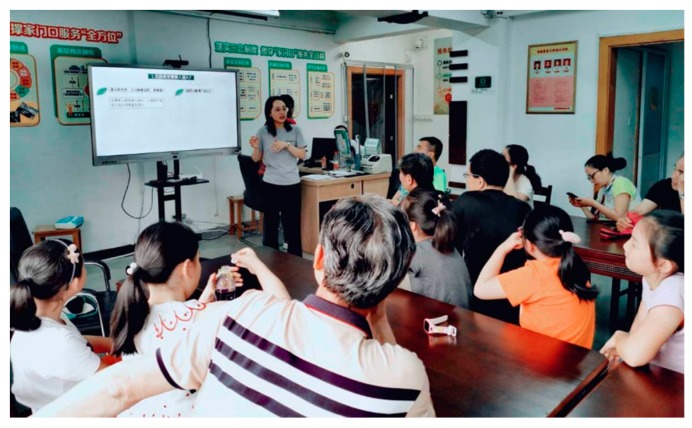
The workshop for community leaders.

The leaders who are exceptionally active and influential play important roles in community participation in the garden building activities. In 2017, we held a community garden training workshop with the government of Puxing Sub-district in Shanghai to encourage the construction of community gardens by training 34 pioneers in 19 neighbourhoods, mainly consisting of members of neighbourhood committees, and a few of most competent neighbourhood representatives (Figure 5). Some of these leaders planned to start building a community garden after the training, while some had already initiated the construction of a garden but encountered some problems and looked for support from the Sub-district government and professionals. The training covered planning and design techniques, as well as gardening technology. The two-day workshop included indoor lectures, outdoor DIY training practice, and site visits to good cases.

After returning to their neighbourhoods, the community leaders then trained the residents to apply knowledge learnt from the workshop in their garden buildings. Three sessions of such workshops have already been carried out. This method, which focuses on training community leaders and providing technical support, significantly increases the speed of propagation of the Community Garden Initiative.

#### 2.3.4. Community’s Independent Proposal, Construction, and Management: Local Co-Creation Team

**Figure 6:**
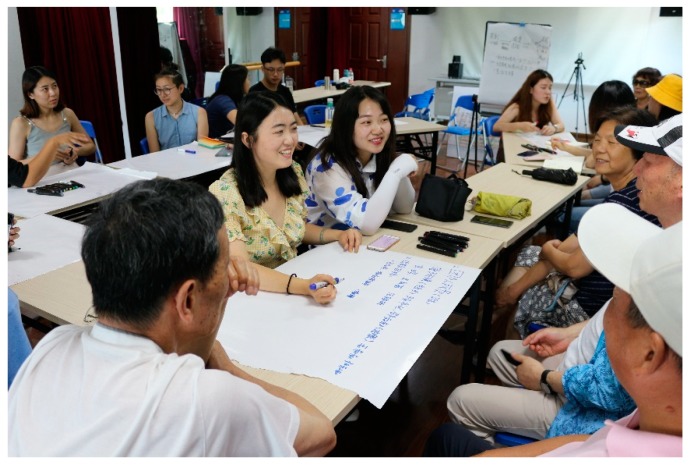
Collective design of residents and students.

In 2019, we initiated an on-site collective design scheme, which was named Local Co-Creation Team (LCCT). This action mobilized residents to propose plans of community garden to improve their neighbourhood environment; meanwhile, engaged young students into the activities by practising their professional skills. At the same time, LCCT allows residents’ requests to be heard by the entire society via academic communities represented by universities. According to the plan, both undergraduate students and the group of residents can propose the theme, location, scale, and scheme on their own (Figure 6). When the proposals respectively from the students and from the residents happen to match the needs of each other, the two parties can form a group to implement the plan. T University provides funding for basic construction materials. The group proposals are not limited to community gardens but expand to various projects related to local environments and culture. For example, some proposals are relating to the building of facilities like leisure benches along walls and planting trees and other greeneries; building eco-friendly lighting structures using plastic bottles and collecting discarded household items to create raised beds; establishing a therapy garden; painting murals on walls; and looking for vacant lots for reuse. These proposals reflect residents’ most practical and urgent needs. The group also absorbs more residents from the community to expand its scale and influence and to collectively construct, maintain, and monitor the garden.

**Figure 7:**
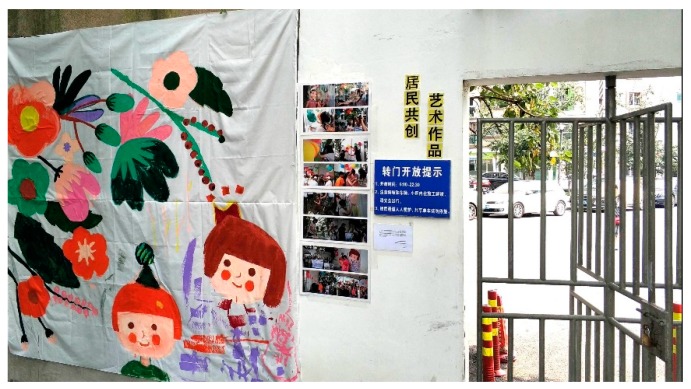
An art activity of the ‘Door of Harmony’.

Among more than ten proposals, the proposal ‘The History of the Disappearance of the Wall’ reminds the public of the magic door in KIC case. The proposal plans to make a documentary about the magic door’s origin and current status and to facilitate the door’s continuous design and maintenance (Figure 7). From proposals to the establishment of the group, the whole process demonstrates the project’s inclusiveness of the community.

#### 2.3.5. Social Initiative with Researchers’ Instructions: Self-Seeding Plan

These efforts to develop the Community Garden Initiative have received attentions from all over the country, in addition to online inquiries from numerous individuals and groups. The research team then started the Self-seeding Plan in 2018, which encompasses a series of plans aiming to provide guidance to the individuals and groups who are interested in constructing community garden and equipping them with necessary knowledge to take the first step towards the garden building. Cooperating with a non-governmental organisation, we enacted the Self-seeding Plan through online Q&A sessions and offline trainings. Three sessions have now taken place involving sustainable planting techniques, ecological garden design, and garden construction plans with professional knowledge. Trainees from more than ten provinces have attended these sessions. By cultivating these citizens who are fond of gardening, we encouraged them to influence more residents in their communities to voluntarily join in constructing gardens, forming growing on-site social organisations. The participants embraced the broader goal to create a better environment and to achieve community cohesion.

Through the compiling online and offline Q&As and combining practices from other projects as mentioned above, the research team published the *Create a Beautiful Homeland Together: Pamphlet for Community Garden’s Practice*, which demonstrates the types of community garden; preparatory surveys that should be undertaken before construction; potential difficulties; bullet points of design, maintenance, and management; and self-governed group-building. The pamphlet illustrated in lively and clearly designed images and concise language, which is easy to understand and to promote the dissemination of community garden building in society.

### 2.4. Questionnaire Surveys and In-Depth Interviews

An integral part of community-engaged participatory research is the inclusion of community members in the interpretation of results [9,41]. After five years of the implementation of the community garden initiative, in July 2019, we distributed a questionnaire survey conducted in-depth interviews that aimed to identify the initiatives’ influence on individuals and the neighbourhoods. The questionnaire survey contained two dimensions: the participants’ comments on the health status of individuals, neighbours, and the situation of the physical areas before and after participation; and the participants’ understanding of and attitude towards the community garden project (Table 1 and Table 2, Appendix A) [42]. The interviews were taken place to explain and supplement after the questionnaire survey. 

The research team published questionnaires on the website of Wenjuan.com, then shared the link in the WeChat groups (a social media prevalent in China) of the participants in five representative activities. The completed surveys were collected online in anonymity two days later. The following table showed the number of received effective questionnaires and the amount of the participants as the targeted population (Table 3). The community garden actions benefit residents in the neighbourhoods directly, with a population size of approximately 3000 to 4000 per neighbourhood.

**Figure A1:**
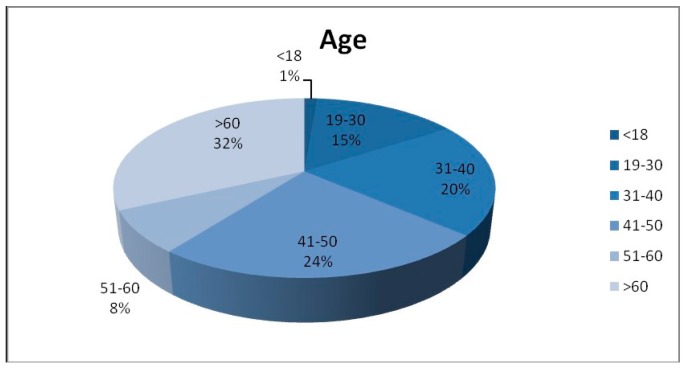
Age.

**Figure A2:**
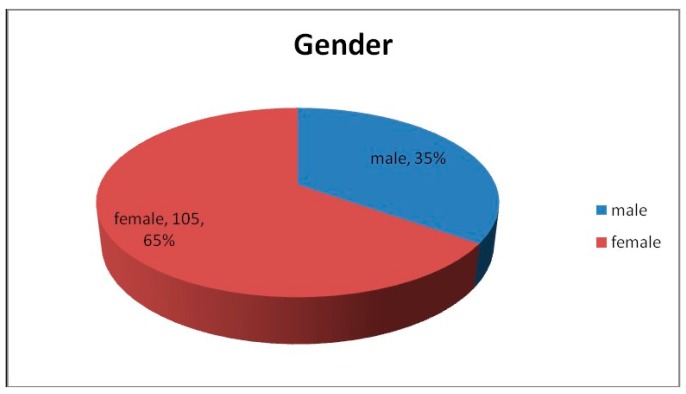
Gender.

**Figure A3:**
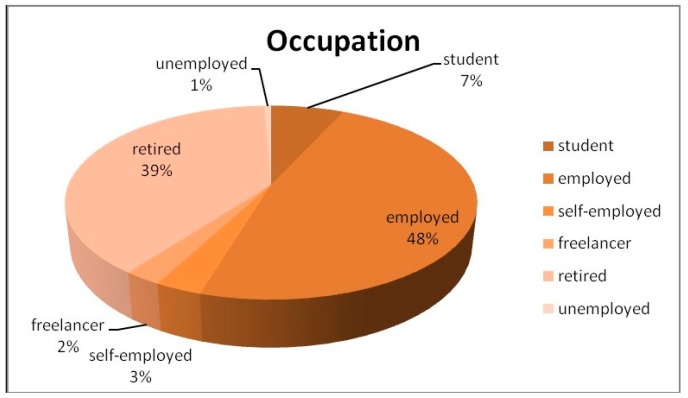
Occupation.

**Figure A4:**
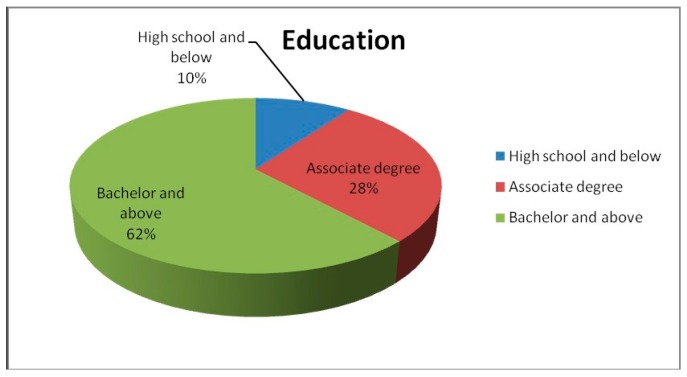
Education.

**Figure A5:**
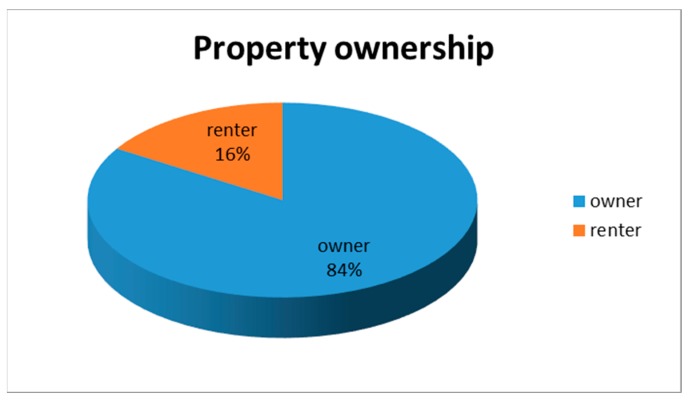
Property ownership.

Five sample projects received 161 effective questionnaires in total. The graph allows us to profile the social attributes of the participants in the five cases (Appendix B, Figure A1, Figure A2, Figure A3, Figure A4 and Figure A5). Overall, the distribution of age is relatively large and even, in accordance with the ageing demographic status in Shanghai (by the end of 2018, the senior population in Shanghai was 5.0328 million, 34.4% of the total registered population [43]). The number of young people under 30 is relatively low; the corresponding proportion of students is also relatively low. The number of females is almost twice that of males. People with stable occupations and retirees constitute most of the data. The proportion of self-owned housing is extremely high, at 84%.

The Herb Garden was the first experiment of the Community Garden Initiative. The government was also in the embryonic stage of encouraging communities to adopt self-governing policies. We selected a community with a relatively good governing foundation to launch the garden construction. We conducted the site selection, design and construction, consulting with residents at each step. We also cultivated residents’ horticultural skills and fostered residents’ self-governing capacity to make sure the follow-up maintenance and management of the garden after the project was completed. For KIC Garden, real estate and property management agency was involved in and provided financial support for the first stage of construction. At the same time, an NGO joined the follow-up improvement, operation, and management, with the principle of community engagement. The measure of training community leader in Puxing Sub-district motivated competent personnel to mobilise more residents to join garden building, facilitating the community garden’s propagation and self-governing cultivation. The LCCT was designed to foster autonomous garden building activities; thus, the research group intentionally stepped back from providing guidance for the project. The Self-seeding Plan mainly provided technical support and training for people who intended to launch garden-building activities by themselves. It intended to break geographical restrictions and extend the Initiative into a much wide scale.

## 3. Results and Discussion

### 3.1. Rapid Development of People’s Participation in Community Garden Building

The five actions to develop community-based garden building demonstrated a progressive process with decreasing interventions from the outside (Table 4). In the first phase, researchers took the initiatives and played a leading role in guiding residents’ involvement step-by-step. In the second phase, enterprises and NGOs were mobilized to participate in community garden building together with residents. The third phase is to foster community leaders as pioneers to guide residents’ participation. In the fourth phase, communities are expected to independently carry out garden building activities; and the fifth phase is to develop the Initiatives into a public campaign towards the whole society with the assistance of researchers.

As the research moving forward, the participants expanded from the nearby community of T University to other districts in Shanghai, and then to the whole country, with overlapping time frames (Table 4). The residents’ involvement also progressed from a relatively small scale to the fullest extent. This started with the partially participation in construction and daily maintenance and management in the Herb Garden and KIC garden cases, followed by the guidance provided through community leaders training in Puxing’s Sub-district for residents who were engaged in the design and construction process of neighbourhood gardens, then LCCT’s autonomous proposal and planning, reaching the independent initiative of residents in the Self-seeding Plan.

**Figure 8:**
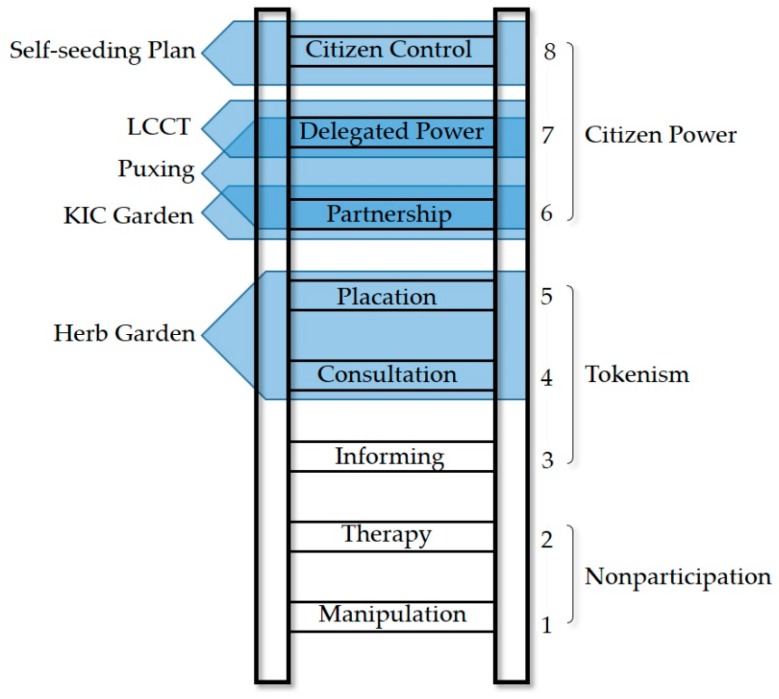
The ladder of community participation.

The corresponding participation level of each action is reflected in Sherry Arnstein’s ladder of participation in the following image (Figure 8): the Herb Garden’s opinion consultation and adoption by professionals can be seen as the fourth and fifth rungs of ‘consultation’ and ‘placation’; the co-construction by the company, NGO, and residents in KIC Garden belongs to the sixth rung of ‘partnership’; training for community leaders and the leaders guiding communities’ construction in the Puxing case is a transitional phase, falling into the sixth and seventh rungs of ‘partnership’ and ‘delegated power’, respectively; LCCT’s decision of the plan fits the seventh rung of ‘delegated power’; and the Self-seeding Plan is elevated to the highest rung.

The rapid increase of community gardens, the quick expansion in a vase geographical scale, and the fast development of their management models within the past five years have reflected the society’s quick understanding and acceptance of participation in community public affairs.

### 3.2. Measures to Increase Community Participation

We applied multiple measures to increase communities’ involvement in the garden building activities:

(1) Cultivating community teams for self-governance. One of the criteria that indicate a community garden’s maturity is whether it has formed organisations with self-governing abilities [44]. The Herb Garden is a representative using this method. The horticultural interest group in the Herb Garden transformed into a self-organising community team after five years of operation. With more residents involved in the team, rules have been established to manage and regulate members’ responsibilities and duties. The group’s self-management operations have now become mature, not only conducting maintenance and management independently but also promoting and exemplifying models to the surrounding communities’ garden building. Comparing the participatory data from the questionnaire, the Herb Garden received the highest score in terms of enthusiasm for community participation and devotion (Table 2, Q6).

(2) Organising diverse activities based on the garden to create a shared space with an inclusive atmosphere. For this reason, we designed a moveable architecture in the KIC Garden. The NGO involved in the management of the KIC Garden developed both indoor and outdoor activities such as a reading club, academic lectures, parent-child cooking, and nature education to attract people of different ages, occupations, and interests. Through these activities, the intention of building such a garden into space ‘by and for all’ in the first place has been realised, and environmental equity is promoted.

(3) Looking into local culture to build community identity. Exploring the cultural characteristics and residents’ collective memory of their community creates a sense of belonging for residents, making them more enthusiastic about community events. The design and construction communication process in the LCCT groups helps explore local culture and residents’ common concerns, through which residents get to know their neighbourhood better and actively participate in community public affairs.

(4) Disseminating and promoting research outcomes effectively. The experiment of Puxing indicated that raining community leaders to provide instructions for the community garden building is an effective method of propagation. The published brochure for the community garden’s practice in the Self-seeding Plan is another successful example for spreading the research outcomes. The brochure includes diagrams with concise language that can be understood by residents at all ages.

### 3.3. Comparison of Participants’ Feelings among Five Cases

Contrary to the progressive increase of resident participation in each action, participants’ subjective feelings in the five case generally turned negative. According to the questionnaire statistics, the differences between ‘before’ and ‘after’ are decreasing from the Herb Garden to the Self-seeding Plan (Table 1). That is to say, with the improvement of empowerment to residents, as well as the reduction of interventions from the outside, the degree of satisfaction among residents about the effects of the community garden project declined.

We further interviewed participants in response to this contradictory phenomenon. Participants in the Herb Garden and KIC generally stated that each stage of the projects was well-organised and led by experts and neighbourhood committees, with their opinions respected and adopted. They felt encouraged, relaxed, and the atmosphere was harmonious in collective activities. However, in the Puxing, LCCT, and Self-seeding Plan, the participants somehow experienced a sense of uncertainty regarding their gardening skills to complete the project and felt stressful regarding the organisation. This indicates that these groups still rely on the organisation from the outside as they used to. When granted with a higher degree of decision-making power and lower external interventions, the participants showed lower satisfaction for the experience. Mr Li, one of the community leaders in Puxing Sub-district, said that he had thought it was simple in the training class, but when he returned to his neighbourhood to act as a leader in horticultural activities, he encountered various unexpected situations in term of the organization. Some neighbours wanted the garden to be as close as possible to their homes while others preferred to be in distance to avoid noise. A neighbour asked if they could enjoy certain privileges because they might contribute more than those who won’t involve in gardening. Some people suggested that regulations on long-term maintenance should be established before the garden was constructed. Although he felt that many of the questions were interesting and inspiring, he felt stressful in those days. The Herb Garden project was carried out under the guidance of researchers and the local government when self-governance at the community level was just in the initial stages. After two years of operation, it gradually stepped into a self-governing state. The differences in impressions before and after participation ranked the highest in this survey. In the interview, when looking back to the garden’s development in the past five years, the residents present a clear sense of pride.

### 3.4. Comparison of the Impact of Participation on a Healthy Environment 

**Figure 9:**
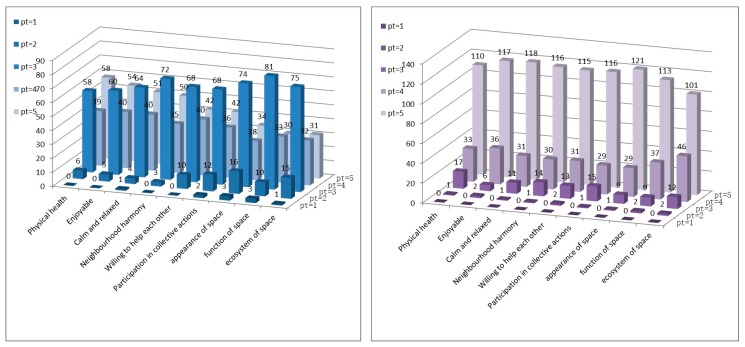
*Score distributions of the Health Status* (**a**) Before participation (**b**) After participation.

The score distributions of all the participants’ perceptions of personal health, neighbourhood health, and physical environment health before and after participation in five cases are shown in the following graph (Figure 9).

According to the Weber-Fechner Law, the difference threshold is logarithmically proportional to the actual objective variable. Therefore, in the statistics of analysing objective changes through subjective feelings, the full score rate, that is, the ratio of the upper threshold, reflects the influence of changes in objective conditions precisely. Taking full score rate into account, the rate of physical health (the number of respondents scoring 5 divided by the number of questionnaires of 161) increases from 36% to 68%, two items of mental health increase from 33%/31% to 73%/72%, three items of neighbourhood health increase from 31%/26%/26% to 72%/71%/72%, and 3 items in environmental health increase from 22%/19%/20% to 75%/70/63% (Figure 9). Therefore, the statistics indicate that participating in a community garden benefits people’s environmental health the most, followed by neighbourhood and mental health experience, and finally personal physical health experience.

### 3.5. Participants’ Attitudes towards the Community Garden Building

**Figure A6:**
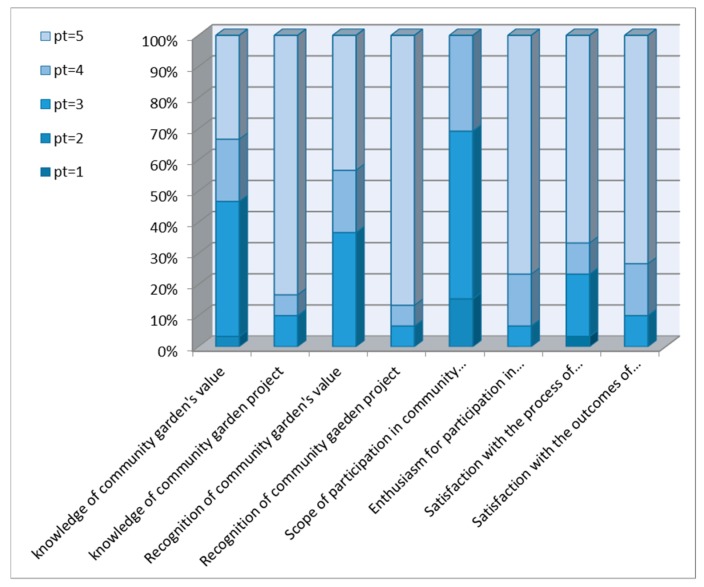
The Herb Garden.

**Figure A7:**
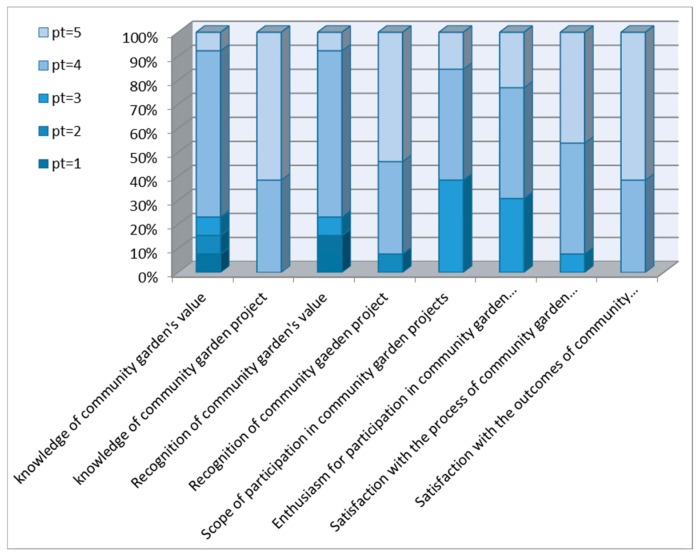
The KIC Garden.

**Figure A8:**
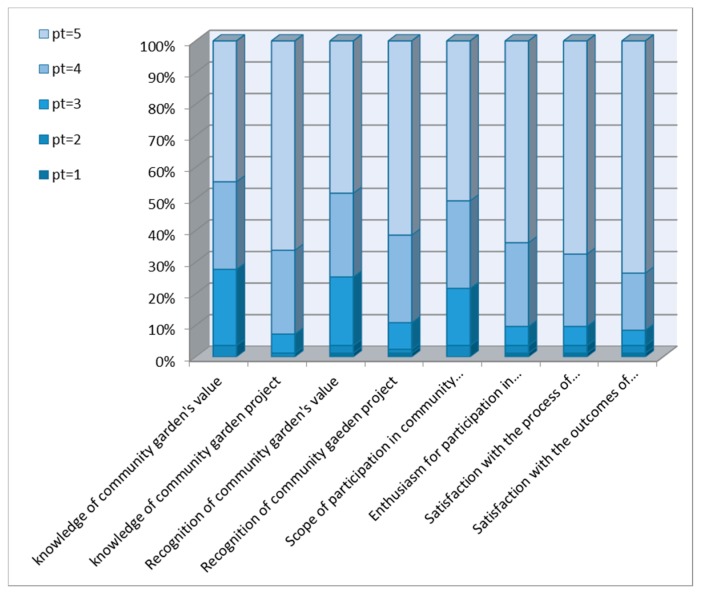
Puxing Sub-district.

**Figure A9:**
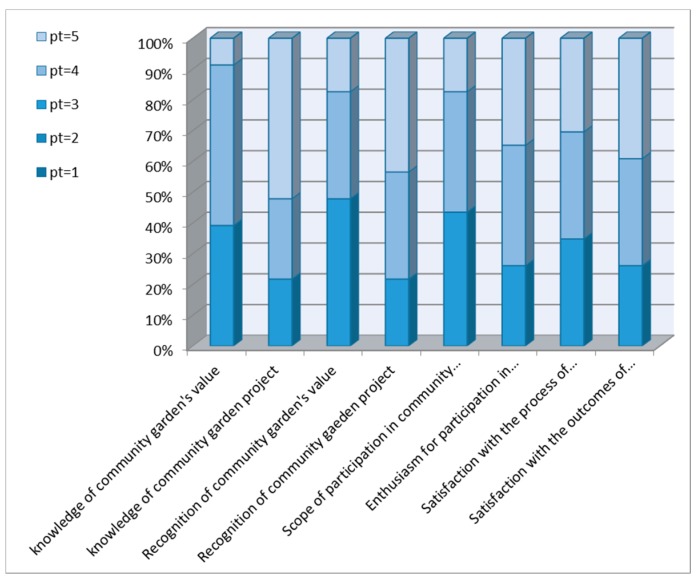
The LCCT.

**Figure A10:**
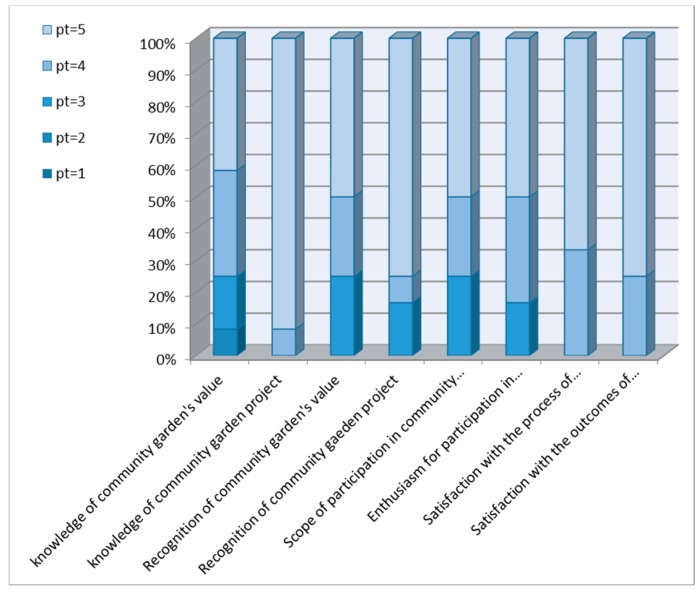
The Self-seeding Plan.

Comparing the scores on the eight sub-factors that demonstrate of participant’s attitudes towards the community gardens projects (Table 2, Appendix B, Figure A6, Figure A7, Figure A8, Figure A9 and Figure A10), we can see that in every action, the recognition score of the community garden’s value (Q3) is higher than the awareness score (Q1). However, the opposite result takes place in the score of the community garden project, for which the awareness score (Q2) is higher than the recognition score (Q4). The relationship between the two groups of factors reflects that, although residents tend to have a relatively high acceptance of scientific concepts, they become discreet and concerned when the matter is related to their own environment. The scope and depth of participation (Q5, Q6) also show a generally positive correlation with satisfaction (Q7, Q8), which reveals that people are always positive about the recognition of their own behaviours. Care for the projects and affirmation of participation provide the basis for implementing community-based research starting from the community garden.

## 4. Conclusions

The community-engaged research process begins with a given phenomenon that plays an important role in the community, and the community identifies, analyses, and solves problems. The ultimate goal is to “integrate knowledge gained with action to benefit the community involved” [41]. This study implemented various methods of community-engaged research in healthy environment building using cases of community garden construction.

Through this research, a cooperative network of community gardens for research and practice has been formed, based on community-university partnerships linking government, enterprises, NGOs, and self-governing community groups. In particular, we conducted service-learning and achieved the goal of two-way participation of community-university partnerships by engaging undergraduates in community garden design and construction. These community garden activities have gained recognition through society and acquired governmental support; they have also facilitated the development of NGOs in China.

The six principles of CBPR suggested by NIEHS, mentioned in the Introduction section have been employed in this study. First, we use multiple measures such as building community team and the organising diverse activities to promote positive collaborations and encourage participation in each phase of the experiments. Second, we foster co-learning between researchers and communities through which residents acquire skills to design, construct and manage garden while researchers better understand the practical needs as well as various creative ideas of the community in garden construction. In addition, students practice what they have learned at school. Third, residents’ common concerns regarding the garden are fully respected in all cases, and in some cases such as the LCCT and the Self-seeding Plan, researchers employed community-driven approaches. Fourth, we have compiled a concise brochure to explain the steps and skills required for community garden building as an effective way to disseminate research results. Fifth, we create an inclusive environment by encouraging residents at all ages, from different occupations, and educational backgrounds to become involved and explore the features of their communities. Sixth, we recreate community identity by connecting residents from other neighbourhoods, organisations and universities through the garden building, as what has been done in the KIC garden.

This study also supplemented the third principle of ‘ensure projects are community-driven’ of the CBPR methods. According to a comparison of participants’ feelings towards the project’s effects, the community-driven principle is hard to apply or may not bring noble experiences in communities where the basis of self-governance is relatively weak. Often, in the initial stage of community-building, residents tend to request for their own sake; thus it is difficult to identify common concerns. Moreover, some residents lack the motivation to participate and expect the government or other parties to arrange everything for them. Therefore, two more points are necessary to supplement the principle of ‘community-driven’. First, capacity-building should be emphasised in the preliminary phase of self-governance, where external interventions are essential to boost local enthusiasm and confidence among the residents. Second, interventions from the experts or other organisations should be adjusted and adaptive to changes of self-governing capacities. That is, when the community’s ability to self-governing is weak, external support for management and guidance need to be comprehensive and strong; while with the strengthening of the capacity, external forces should step back to allow the community to develop at its own wills. Taking KIC Garden as an example, at the start of the project, the old neighbourhood lacked enthusiasm while the new one had no clear requests. Thus, the design and construction work was dominated by researchers and the enterprise in the initial phase. During the construction and maintenance process, residents in the two neighbourhoods were linked together through various activities, fostering residents’ enthusiasm for participation, which even attracted and involved people living farther away. Along with the improvement of the garden, many activities were directly initiated by residents, with minimum technical external interventions from the professionals.

The improvement in the community’ environmental health is evident when vacant barren plots are transformed one after another into gardens shared by residents with aesthetic, ecological, and social functions. The increased social exchange among neighbours and people’s proximity to nature promoted the harmonious neighbourhood relations, community cohesion and residents’ mental health. Responding to this result, we are now launching a series of Healing Garden experiments to provide auxiliary environmental treatment for people with autism, depression, and other mental problems. Six communities in Shanghai have joined the experiments.

According to the result of the surveys on residents’ attitudes, the community garden, rooted in the daily life of the community, is one of the least controversial issues among neighbourhood public affairs. Promoting residents’ interaction through the construction and management of a garden can activate residents’ enthusiasm for public issues and engage their sense of community. The community-engaged research methods in this study apply not only to community gardens but also to spatial rehabilitation, healthy environment building, and community affairs in other areas. In the absence of an officially registered system for community planners in China, the government from the district where the university is located was driven by these initiatives to conduct registered community planner system starting in 2018. Several Professors in T University have been employed as community planners for community planning and environment micro-regeneration.

The Community Garden Initiative has been rapidly promoted by universities, communities and the government of Shanghai, but the practice is still not fully popularised or conducted nationwide. Although the above five actions from Shanghai represent a momentum of this growing trend, samples from other cities need to be included in further research. In addition, distributing questionnaires for the five cases at the same time had certain limitations, because the timing of each case varies from 2014 to 2019 while the impressions and attitudes of participants often improve with the continuous evolution of the gardens. Another limitation exists in the weak analysis of garden’ impact on environmental health, but this study focuses more on the facilitation of healthy environments through participating in community garden activities, as well as the application and reflection on participatory research methods. As leading experimental research with community participation in the field of community garden building, further study will be needed to focus on more detailed discussions of community-engaged research methods.

## Figures and Tables

**Table 1:** Participants’ impressions of health status before and after participation.

Factor		Individual Health Status			Neighbourhood Health Status			Physical Environment Health Status		
Sub-factor		Physical health	Mental health		Neighbourhood harmony and inclusiveness	Community cohesion		Aesthetics of the garden	Spatial and ecological functions	
Reasons for factor selection		Measure physical health status before and after participation	Measure mental health status before and after participation		Measure neighbourhood relationships before and after participation	Measure changes in community cohesion before and after participation		Measure changes in appearance of the garden before and after participation	Measure changes in community garden’s spatial and ecological function before and after participation	
Question number in questionnaire		Q1	Q2a	Q2b	Q3	Q4a	Q4b	Q5	Q6a	Q6b
Averaged difference (‘after’ minus ‘before’)	Herb Garden	1.8	1.6	1.4	0.7	1	1.1	0.9	1.1	1.3
	KIC	1.7	1.6	1.8	0.7	0.8	1.2	1	1.1	1.2
	Puxing	1.1	1.1	1.1	0.7	0.8	0.8	0.8	0.8	0.9
	LCCT	1.2	1.1	1	0.5	0.7	0.6	0.8	1	1
	Self-seeding	1	0.9	0.7	0.4	0.3	0.6	0.7	0.8	0.9

**Table 2:** Participants’ cognition and attitude towards community garden projects.

Factor		Awareness		Recognition		Participation		Satisfaction	
Sub-factor		Knowledge of community garden’s value	Knowledge of community garden project	Recognition of community garden’s value	Recognition of community garden project	Scope of participation in community garden projects	Enthusiasm for participation in community garden projects	Satisfaction with the process of community garden projects	Satisfaction with the outcomes of community garden projects
Reasons for factor selection		Measure understanding of community garden’s impact on health	Measure understanding of the project purposes, contents, and execution process	Measure recognition of community garden’s impact on health	Measure recognition of project purposes, contents, and execution process	Measure phases and contents of participation in community garden projects	Measure enthusiasm for participation in community garden projects	Measure satisfaction with the organisation and execution process of community garden projects	Measure satisfaction with the outcomes of community garden projects
Question number in questionnaire		Q1	Q2	Q3	Q4	Q5	Q6	Q7	Q8
Average score (Based on questionnaire Statistic)	Herb Garden	3.8	4.8	4.1	4.8	4.2	4.7	4.4	4.6
	KIC	3.6	4.6	3.6	4.4	3.8	3.9	4.4	4.6
	Puxing	4.1	4.6	4.2	4.5	4.3	4.5	4.5	4.6
	LCCT	3.7	4.3	3.7	4.2	3.7	4.1	4.0	4.1
	Self-seeding	4.1	4.9	4.3	4.6	4.3	4.3	4.7	4.8

**Table 3:** Data of the five actions.

Action	Neighbourhood Population	Participant	Effective Questionnaire	Percentage of Questionnaires to Participants
Herb Garden	4000 in old neighbourhood	150	30	20%
KIC Garden	3500 in old neighbourhood; 3000 in new neighbourhood	80	13	16%
Puxing District	19 old neighbourhoods; 3000 residents per neighbourhood	700	83	12%
Local Co-Creation Team	2 old neighbourhoods; 3000 residents per neighbourhood	50	23	46%
Self-seeding Plan	Neighbourhoods in 11 Provinces	95	12	13%

**Table 4:** Five phases of community garden initiative.

Number	Phase	Total Quantity of Projects	Starting Time
1	Researchers’ initiative with community involvement	45	2014
2	Co-construction by enterprises, NGOs, and residents	3	2016
3	Fostering community leader to drive community participation	10	2017
4	Community’s independent proposal, construction, and management	1	2019
5	Social imitative with researchers’ instructions	11	2018

## Appendix A. Questionnaire for Residents Participating in the Community Garden Initiative

### Appendix A.1. Basic Information

1. Age: [A] Under 12 [B] 12-17 [C] 18-24 [D] 25-40 [E] 41-60 [F] Over 61
2. Gender: [A] Male [B] Female
3. Occupation status: [A] Student [B] Employed [C] Freelancer [D] Self-employed [E] Retired
4. Education: [A] High school and below [B] Associate degree [C] Bachelor and above
5. Property ownership: [A] Owner [B] Tenant

### Appendix A.2. Health Status before and after Participating in the Community Garden Projects

How do you feel about the following situations before and after participating in the construction of the community garden? Please rate them according to the extent from 1 to 5: ‘1′ (very bad), ‘2′ (relatively bad), ‘3′ (average), ‘4′ (relatively good), ‘5′ (very good).

**Item**	**Question**		**before Participation**	**after Participation**
Your own health status	Q1. Physical health status			
	Q2. Mental health status	Q2a. I think I am helpful to others, and my life is enjoyable.		
		Q2b. Most of the time I feel calm and relaxed.		
Neighbourhood health status	Q3. Neighbourhood harmony and inclusiveness			
	Q4. Community Cohesion	Q4a. Residents are willing to help each other when facing difficulties.		
		Q4b. Residents actively participate in collective actions.		
Spatial environment health status	Q5. Community garden’s appearance			
	Q6. Community garden’s Spatial and ecological functions	Q6a. Spatial functions for learning, entertainments and socialisation activities.		
		Q6b. Ecosystem such as purification of water and air, plants and insects.		

### Appendix A.3. Cognition and Attitude towards Community Garden Projects

What is your evaluation regarding the community garden project? Please rate them according to the extent from 1 to 5: ‘1′ (very low), ‘2′ (relatively low), ‘3′ (average), ‘4′ (relatively high), ‘5′ (very high)

**Item**	**Question**	**Rating**
Awareness	Q1. Your understanding of community gardens’ impact on health.	
	Q2. Your understanding of the purposes, contents and execution of community garden projects.	
Recognition	Q3. Do you agree with these impacts on health brought by community garden?	
	Q4. Do you agree with the purposes, contents and execution of the community garden projects?	
Participation	Q5. The scope of your participation in different phases and sessions	
	Q6. The extent of your enthusiasm for participation in community garden construction.	
Satisfaction	Q7. Satisfaction with the organisation and execution process of the community garden project.	
	Q8. Satisfaction with the outcomes of the community garden project.	
